# COP Cells and Tissue Loss Syndromes: Frailty, Sarcopenia, and Osteoporosis

**DOI:** 10.1093/geroni/igab046.168

**Published:** 2021-12-17

**Authors:** Gustavo Duque

**Affiliations:** The University of Melbourne, St Albans, Victoria, Australia

## Abstract

COP cells have been identified as having a potential role in the pathogenesis of tissue loss syndromes such as osteoporosis and frailty. This is based on the hypothesis that their dysregulation may cause a decrease in bone and muscle formation, which also increase the risk of adverse outcomes such as frailty and disability. Whereas high numbers of COP cells have been associated with osteoporosis and fracture healing, a low percentage of COP (%COP) cells have been associated with frailty and disability. In addition, low expression of lamin A (a protein of the inner nuclear envelope) in COP cells has also been associated with frailty and disability in older persons. In this session, the evidence on quantification methods for COP cells in clinical settings and the potential clinical use of COP cells in tissue loss syndromes will be discussed. This discussion will include current evidence supporting the use of COP cells as a biomarker or as a novel therapeutic approach to these age-related conditions.

